# Loss-of-function of the long non-coding RNA A830019P07Rik in mice does not affect insulin expression and secretion

**DOI:** 10.1038/s41598-020-62969-x

**Published:** 2020-04-14

**Authors:** Claudiane Guay, Baroj Abdulkarim, Jennifer Y. Tan, Gilles Dubuis, Sabine Rütti, David Ross Laybutt, Christian Widmann, Romano Regazzi, Ana Claudia Marques

**Affiliations:** 1 Department of Fundamental Neurosciences, University of Lausanne, Lausanne, Switzerland; 2 Department of Computational Biology, University of Lausanne, Lausanne, Switzerland; 3 Department of Physiology, University of Lausanne, Lausanne, Switzerland; 4 Present Address: Centre Européen d’Etude du Diabète, Strasbourg, France; 5 Garvan Institute of Medical Research, St. Vincent’s Clinical School, UNSW Sydney, Sydney, New South Wales Australia

**Keywords:** Non-coding RNAs, Metabolism

## Abstract

Long non-coding RNAs (lncRNAs) contribute to diverse cellular functions and the dysregulation of their expression or function can contribute to diseases, including diabetes. The contributions of lncRNAs to β-cell development, function and survival has been extensively studied *in vitro*. However, very little is currently known on the *in vivo* roles of lncRNAs in the regulation of glucose and insulin homeostasis. Here we investigated the impact of loss-of-function in mice of the lncRNA A830019P07Rik, hereafter P07Rik, which was previously reported to be associated with reduced plasma insulin levels. Compared with wild-type littermates, male and female P07Rik mutant mice did not show any defect in glycaemia and plasma insulin levels in both fed and fasted state. Furthermore, P07Rik mutant mice displayed similar glucose and insulin levels in response to an intra-peritoneal glucose tolerance test. *Ex vivo*, islets from mutant P07Rik released similar amount of insulin in response to increased glucose concentration as wildtype littermates. In contrast with previous reports, our characterization of P07Rik mouse mutants revealed that loss of function of this lncRNA does not affect glucose and insulin homeostasis in mice.

## Introduction

Long non-coding RNAs (lncRNAs) are a heterogeneous class of transcripts loosely classified as being longer than 200 nucleotides and lacking protein-coding potential^1^. Gene expression regulation by lncRNAs have been implicated in diverse biological processes in health and disease^2–8^, including diabetes^9–11^.

Diabetes is a group of metabolic disorders characterized by chronic hyperglycaemia due to pancreatic β-cells failure and consequent inability to secrete the appropriate amount of insulin to meet the needs of the organism. Insulin is central to the maintenance of glycaemia by favouring glucose uptake and utilization by the insulin-sensitive tissues. Only fully differentiated β-cells can adequately produce and secrete insulin in response to various nutrients. This feature is acquired postnatally and maturation is completed when pups are weaned in rodents^12,13^. Under certain physiological conditions, like pregnancy and obesity, target tissues become resistant to the action of insulin. To compensate resistance and normalize blood glucose levels pancreatic β-cells mass or activity is increased^14^. Defects in maturation, compensation, and/or survival of the β-cells lead to development of diabetes mellitus.

Hundreds of lncRNAs have been identified in human and mouse pancreatic islets, with most being specifically expressed in insulin-secreting β-cells^15–18^. The expression of some of these β-cell specific lncRNAs is regulated by extracellular glucose concentration indicating they are part of the gene regulatory network controlling glucose and insulin homeostasis^15^. Consistent with the contribution of lncRNAs to diabetes their expression has been found to be dysregulated in the islets of diabetic human donors and animal models of diabetes. Furthermore some lncRNAs map to genetic loci associated with increased susceptibility of developing diabetes^15,16,19–21^. While the precise function of most β-cell-associated lncRNAs remain unknown, some efforts have been made to characterize a limited number of candidate lncRNAs and to determine their role in β-cell development, maturation and function^16,19,21–24^. So far, only the role of βlinc1, the mouse orthologue of the islet-specific human HI-LNC15^15^, has been established i*n vivo*^23^. Deletion of βlinc1 in mouse led to a 50% reduction in β-cell mass at birth and to impaired insulin release in response to glucose in adult animals.

We aimed to expand the repertoire of lncRNAs implicated on glucose homeostasis. For this reason, we turned our attention to animal models generated by the international knockout mouse consortium (IKMC)^25^. While the priority of the IKMC is to study protein-coding genes, some lncRNAs have also been targeted. Mining this publicly available resource, we identified an intergenic lncRNA A830019P07Rik (P07Rik) whose loss-of-function was reported by IKCM to be associated with decreased circulating insulin levels^26^. Interestingly, we found that the expression level of P07Rik was changed during β-cell maturation and in diabetic mouse islets, suggesting that under certain conditions, the lncRNA P07Rik could play a role in the regulation of β-cell function. We therefore investigated the effect of loss-of-function of P07Rik on blood glucose and insulin homeostasis *in vivo*.

## Results and discussion

### P07Rik transcript is conserved in rodents

**Figure 1:**
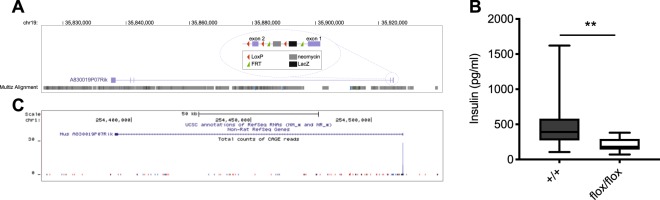
P07Rik knockout is associated with low circulating insulin levels. (**A**) Genome browser view of the region encoding P07Rik (mm10). Exons and introns are represented as purple squares and lines, respectively. Circle represents schematic the approach used by the IKMC to generate P07Rik knockout mice. (**B**) Distribution of non-fasting plasma insulin levels, obtained by the IKMC, in P07Rik mice and control. Data are represented as tukey boxplot showing median, 25^th^ and 75^th^ percentile (**C**) Genome browser view of the orthologous region in rat (rn6) of mouse P07Rik. Mouse exons and introns are represented as blue squares and line respectively. Arrows indicate the direction of transcription. Total CAGE read counts are represented below gene locus, blue and red represent reads produced from the minus and plus strand, respectively.

Through data mining of phenotypes associated with lncRNA loss-of-function mutants generated by the IKCM, we identified an intergenic lncRNA, *A830019P07Rik* (*P07Rik*) reported to display decreased plasma insulin levels. The *P07Rik* locus encodes a multiexonic non-coding transcript (Fig. 1A). *P07Rik* mutants have a floxed promoter-driven neocassette in their first intron (Fig. 1A). This loss-of-function strategy leads to the inclusion of the trapping cassette, resulting in premature termination of P07Rik transcription and constitutive loss of exon 2 expression^25^ (Supplementary Fig. S1).

When compared to control mice (~4000, both males and females), homozygous truncation of *P07Rik* transcription was reported by the IKMC to be associated with a 2-fold decrease in plasma insulin levels in non-fasting 16-week-old female and male mice (14, equally distributed between males and females, Fig. 1B).

We first investigated whether P07Rik locus is conserved in rodents. We identified the *P07Rik* orthologous region in the rat genome and found that the genomic region encoding this lncRNA is partially conserved in rodents (Fig. 1C). We designed several RT-PCR primers specific to the putative orthologous rat *P07Rik* sequence (Supplementary Table S1) and confirmed that the 5′ proximal exon of this gene is expressed in the rat insulin-secreting cell line INS 832/13 (data not shown). In contrast, we found no evidence for expression of the 3′ end exon, suggesting a different *P07Rik* transcript structure in rat. Our results are consistent with previous data on lncRNA conservation in rodents, indicating fast evolution of the expression and structure of these class of transcripts^27^.

### Differential expression of P07Rik in mouse tissue and diabetic islets

**Figure 2:**
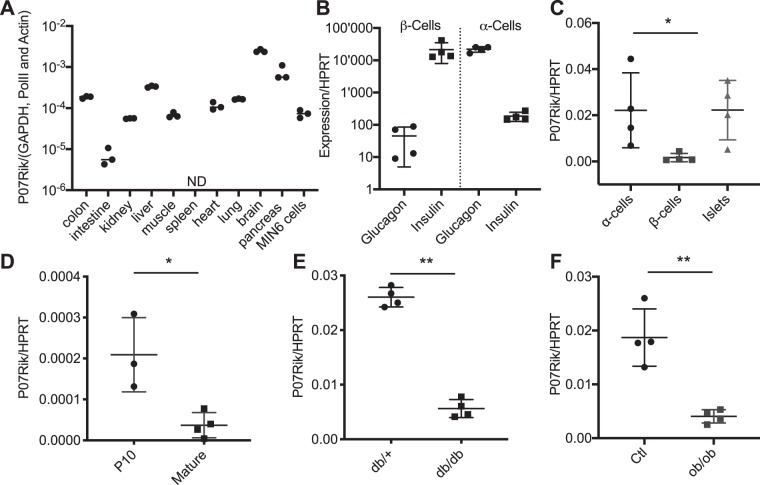
P07Rik is downregulated during development and in obese and diabetic mice. P07Rik’s spatial expression pattern measured by qPCR in (**A**) indicated tissues of adult mice and MIN6 cell line. Glucagon, Insulin (**B**) and P07Rik (**C**) expression in α− and β− cells isolated from 3 month old rats. P07Rik expression in (**D**) rats at P10 and 3 month of age and 13–16 weeks old (**E**) db/+ and db/db mice and (F) +/+ and ob/ob mice. Data is presented as dots indicating each separate sample and the lines indicating the mean ± SD. Abbreviations: PolII-RNA Polymerase II, GAPDH- Glyceraldehyde-3-Phosphate Dehydrogenase, HPRT-Hypoxanthine Phosphoribosyltransferase. Statistics: *p < 0.05, **p < 0.001, two-tailed t-test. “ND” indicates not detected.

Next, we investigated the expression of *P07Rik* in a panel of adult mouse tissues and in the MIN6B1 β-cell line. We found that *P07Rik* is highly expressed in brain but is also detectable, albeit at lower levels, in other adult tissues including pancreas and in MIN6B1 cells (Fig. 2A). To gain initial insights into pancreatic islet cell expression we quantified the expression of P07Rik in pancreatic α− and β− cells (Fig. 2B) and found that in adult rat, P07Rik expression in islets is highly enriched in α-cells (two-tailed t-test *p* < 0.05, Fig. 2C).

Given the reported decrease in insulin levels associated with P07Rik loss-of-function, we next assessed whether this lncRNA is involved in the acquisition of a fully functional islet phenotype during post-natal maturation and/or its dysregulation associated with type 2 diabetes. We found that *P07Rik* expression is significantly increased in immature P10 rat islets compared to fully mature adult islets (Fig. 2D). Interestingly, *P07Rik* expression is significantly decreased in islets of obese and normoglycemic ob/ob mice and of obese and diabetic db/db mice (Fig. 2E,F). These mice do not produce leptin or lack the leptin receptor, respectively, and therefore become progressively obese and insulin resistant. While the ob/ob mice remain normoglycemic, the db/db become diabetic by the age of 12–16 weeks^28^. Taken together these observations suggested a possible involvement of P07Rik in the regulation of pancreatic islet function.

### Loss-of-function of P07Rik does not affect glucose and insulin homeostasis

**Figure 3:**
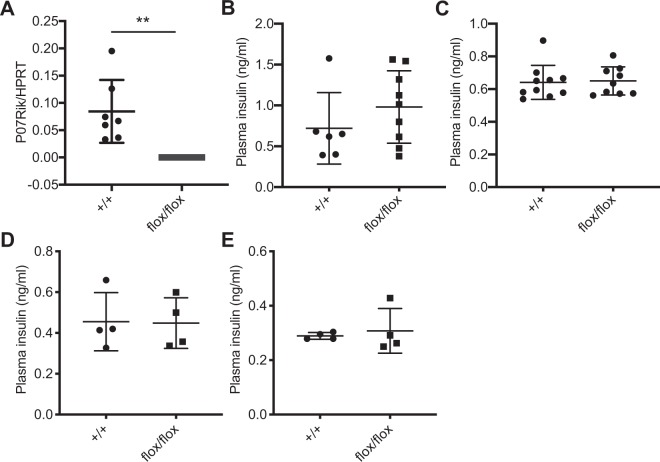
P07Rik flox/flox does not affect plasma insulin levels. (**A**) *P07Rik* relative expression measured by qPCR in islets of WT and flox/flox 12–13 weeks old littermates. Non-fasting (**B,C**) and fasting (**D,E**) plasma insulin levels were measured in 16 weeks old male (**B,D**) and female (**C,E**) mice respectively. Data are represented as dotplot representing each individual animal and the lines indicating the mean ± SD. Abbreviations- HPRT-Hypoxanthine Phosphoribosyltransferase. Statistics: **p < 0.001, NS-p > 0.05, two-tailed t-test).

**Figure 4:**
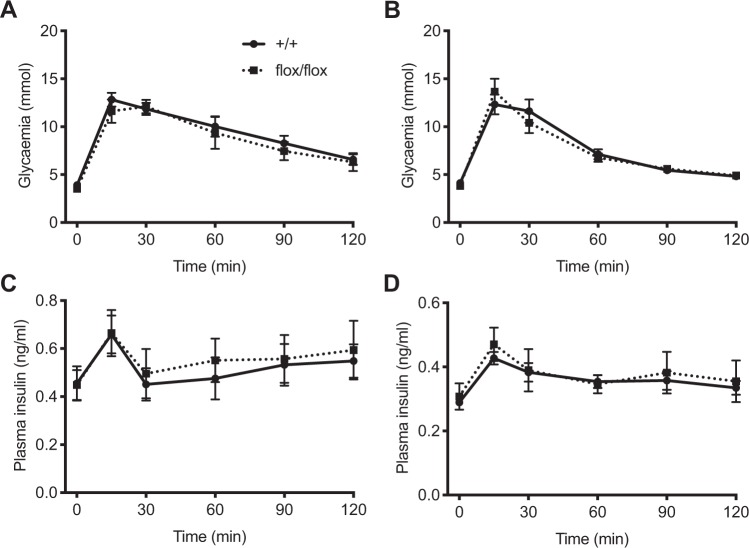
P07Rik flox/flox does not affect glucose sensitivity. Blood glucose levels (**A,B**) and plasma insulin levels (**C,D**) were measured after IPGTT of male (**A,C**) and female (**B,D**) mice at 17 weeks of age. Data are represented as mean ± SE of n = 4 animals. Statistics: **p < 0.001, NS-p > 0.05, two-tailed t-test).

To investigate the role of P07Rik on insulin and glucose homeostasis *in vivo*, we used cryo-preserved embryos of P07Rik heterozygous mice to establish a colony of P07Rik^flox/flox^ mutant (flox/flox) and P07Rik^+/+^ wildtype control (WT) littermates. Downregulation of P07Rik expression was confirmed on isolated pancreatic islets (Fig. 3A) of 12–13 weeks old animals. As shown in Table 1, mutant (flox/flox) and wildtype littermate P07Rik mice display similar body weight and blood glucose levels in fed and fasted state, both in males and females aged 3 and/or 4 months. Since the IKMC reported a decrease in circulating insulin levels in non-fasting P07Rik^flox/flox^ (Fig. 1C), we measured plasma insulin levels in 16 weeks old males and females. In contrast to the data provided by the IKMC, this analysis revealed no difference in non-fasting insulin levels in P07Rik^flox/flox^ mice compared to the reported IKMC data (Fig. 3B,C). We found no significant differences in 16h-fasting plasma insulin levels between the P07Rik female and male mutant and wildtype littermates (Fig. 3D,E). To estimate if our experiment was sufficiently powered, we used the average circulating insulin levels obtained in wild-type animals (0.7 ± 0.2 ng/ml) to estimate the sample size needed to detect a significant (p-value < 0.05) 50% decrease in P07Rik mutants. The sample size used in the present analysis (18) is almost twice the estimated sample size (10) necessary to reproduce a 50% decrease in wild-type circulating insulin levels. To further investigate potential differences in circulating insulin or glucose levels, we performed an intra-peritoneal glucose tolerance test (IPGTT) in 17 weeks old overnight fasted mice. Using this approach, we again observed no differences in glucose sensitivity or insulin response (Fig. 4) for both female and male P07Rik^flox/flox^ mice compared to wildtype littermates.

**Table 1:** Body weight and blood glucose levels in fasted and fed male and female P07Rik mice.

	Male 3 months		Male 4 months		Female 4 months	
	P07Rik^+/+^	P07Rik^flox/flox^	P07Rik^+/+^	P07Rik^flox/flox^	P07Rik^+/+^	P07Rik^flox/flox ^
Body weight (g)	26.3 ± 1.4 (n = 6)	27.5 ± 1.1 (n = 6)	30.5 ± 3.3 (n = 11)	30.5 ± 1.9 (n = 16)	21.6 ± 1.5 (n = 10)	22.3 ± 2.3 (n = 9)
**Glucose (mmol/l)**						
Fed	6.9 ± 1.2 (n = 11)	7.0 ± 0.8 (n = 9)	6.9 ± 0.9 (n = 8)	6.9 ± 0.8 (n = 15)	6.3 ± 0.8 (n = 13)	6.6 ± 0.9 (n = 12)
Fasted	5.2 ± 0.9 (n = 10)	4.7 ± 0.6 (n = 9)	3.9 ± 0.2 (n = 4)*	3.5 ± 0.5 (n = 4)*	4.0 ± 0.3 (n = 4)*	4.0 ± 0.5 (n = 4)*

*Data from IPGTT.

**Figure 5:**
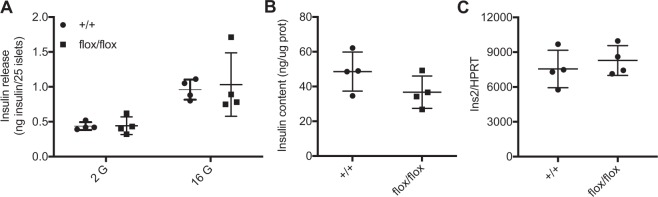
P07Rik deletion does not affect islet insulin secretion, content or expression. Islets isolated from WT and flox/flox littermates were stimulated with 2 or 16 mM glucose and insulin secretion (**A**) and insulin content (**B**) were measured. (**C**) *Insulin2* relative expression was measured by qPCR in islets of WT and flox/flox littermates. Data is presented as dotplot indicating each separate sample and the lines indicating the mean ± SD. Abbreviations: HPRT-Hypoxanthine Phosphoriboslytransferase. Statistics: NS- p > 0.05, two-tailed t-test.

Finally, we measured *ex vivo* insulin content and secretion in response to glucose in pancreatic islets isolated from overnight fasted 3 months old male P07Rik^flox/flox^ and wildtype mice. Again, we found no difference in insulin release at both basal and stimulatory glucose concentrations (Fig. 5A). Furthermore, islet insulin content (Fig. 5B) and *Insulin2* mRNA expression (Fig. 5C) were similar between the two genotypes.

### No detectable changes in islet gene expression upon P07Rik loss-of-function

**Figure 6:**
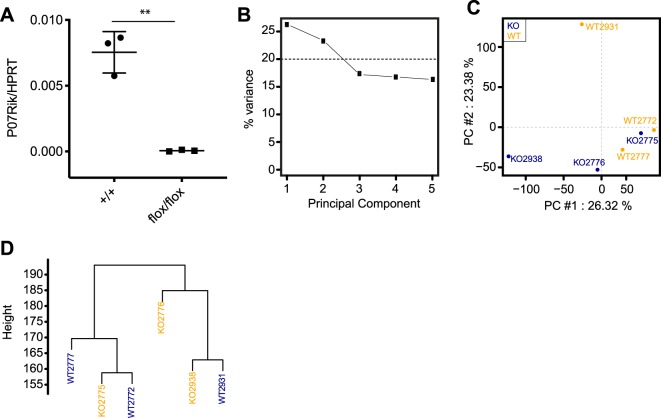
P07Rik deletion results in no detectable islet gene expression changes. (**A**) *P07Rik* relative expression measured by qPCR in islets of WT and flox/flox 12–13 weeks old littermates. (**B**) Elbow plot of RNAseq islet samples showing that principle components 1 and 2 capture the majority of variance in the data. No clear clustering between flox/flox (dark blue) and WT (yellow) samples were observed in (**C**) principle component analysis (PCA) and (**D**) dendrogram of hierarchical clustering analysis.

Given the role of long noncoding RNAs as gene expression regulators, we next aimed to determine if loss of P07Rik function would lead to changes in islet transcriptomes that would support the observed changes in circulating insulin described by the IKMC for these animals. Towards this aim we extracted RNA from the islets of 3 pairs of 12 weeks old male mutants (flox/flox) and litter mate controls and profiled transcriptome wide expression by RNA sequencing (Fig. 6A). Strikingly the transcriptome wide profile of WT and P07Rik^flox/flox^ islets was indistinguishable across all principal components that captures most of the information in the data (Fig. 6B,C) and clustering of samples based on their gene expression did not reveal any differences between genotype (Fig. 6D), indicating that loss of P07Rik did not significantly impacted islet gene expression. Consistent with this differential gene expression analysis revealed that no gene was differentially expressed between mutants and controls (Supplementary Table 2) further supporting that loss of P07Rik has no impact in adult islet homeostasis, under unchallenged conditions.

## Conclusion

Previous studies have identified several lncRNAs in β-cells which are dysregulated during post-natal maturation of insulin-secreting cells or in conditions associated with type 2 diabetes^9–11^. However, so far only very few studies investigated the precise role and function of candidate lncRNAs^16,19,21–24^. Since we are only starting to understand the role of lncRNAs in the regulation of β-cell development, function and survival, more studies, especially *in vivo*, are required to fully appreciate the importance of these transcripts in the maintenance of glucose and insulin homeostasis.

The IKCM aims to accelerate the genetic analysis of mammalian genes through “the generation of a complete resource of reporter-tag null mutations in C57BL/6 N mouse embryonic stem cells (mESC)”^22^. So far, more than 18′500 mouse loci have been knocked down in mESCs by the consortium using a standardized gene trapping approach. About 5′000 of them have been microinjected in blastocysts and their F1 progeny phenotyped^23^. Of this we selected a long noncoding RNA A830019P07Rik because premature termination of this lncRNA was reported to decrease plasma insulin level relative to a large cohort of control mice. Our analysis of this animal model, that instead relies on comparison of mutant animals with littermates revealed that there is no significant differences in plasma insulin levels in both mutant mice. We extended the phenotyping and investigated differences in circulating insulin levels in both fed and fasted state. Furthermore, insulin release in response to glucose *in vivo* or *ex vivo* was not affected. Moreover, islet insulin content and *Insulin2* mRNA levels were identical in the two genotypes.

The most plausible explanation for the divergence between the results obtained in our hands and those from the IKCM is that we used the appropriate wildtype littermate controls. IKCM generated thousands of knockout mice and collected plasma for insulin determination over a long period. This colossal work led to the comparison of plasma insulin levels from 14 mutant (flox/flox) P07Rik mice to the average level of ~4′000 control mice from their cohort. As shown in Fig. 1B, the distribution of circulating insulin measured by IKCM in control mice wide ranging from 200 to 1000 pg/ml. It is therefore difficult to conclude about a difference of plasma insulin levels in a specific cohort of mutant mice. In our study, we used wildtype littermates generated from the same parental heterozygous mice to compare blood glucose and plasma insulin levels in fed state. We did not observe a difference in both female and male of 3 and/or 4 months of age, despite using a cohort of mutant P07Rik animals that is at almost twice the estimated sample size required to detect the previously reported changes.

However, our findings under normal conditions do not rule out the possibility that under other less favorable environmental conditions the mutation of lncRNA P07Rik could affect insulin and/or glucose metabolism. More importantly, β-cell maturation in newborn rats and β-cell failure in a mouse model of obesity-associated with T2D led to an alteration in P07Rik expression, suggesting a possible implication of this lncRNA in the regulation of β-cell development and/or function. The lncRNA P07Rik was also found to be expressed in other tissues regulating glucose homeostasis, including brain, liver and skeletal muscles (Fig. 2A). Therefore, the ability of P07Rik^flox/flox^ mice to maintain glucose tolerance and insulin sensitivity could potentially be impaired under a genetic background favoring obesity or in response to conditions leading to insulin resistance and diabetes development, like a diet rich in carbohydrates and fatty acids. Furthermore, the present analysis does not exclude a potential role of P07Rik in the regulation of gene expression in alpha cells of young animals, where we found this lncRNA to be more highly expressed.

Taken together, our results suggest that under control conditions, P07Rik does not play a major role in the regulation of glucose and insulin homeostasis.

## Methods

### Animal studies

The P07Rik mouse colony was established from cryo-preserved embryos obtained as part of a collaboration with Prof. David Adams (Sanger Institute in Cambridge, UK) by Charles Rivers. The genetic background of these animals is C57Bl/6 N background^29^ so they do not harbor a mutation in the nucleotide transhydrogenase gene^30^. Heterozygous mice were interbred to generate P07Rik^flox/flox^ (mutant) and P07Rik^+/+^ (wildtype) littermates. The mice were genotyped by PCR (see Supplementary Table S1 for primer sequences and Supplementary Fig. S1 for an example of the genotyping results). Both males and females aged 3 and 4 months were used in this study and control mice were C57Bl/6 N P07Rik^+/+^ wildtype littermates. Pregnant female and 3 month-old male Sprague-Dawley and Wistar rats were purchased from Janvier Laboratories (Le Genest-Saint-Isle, France). Offspring of pregnant rats were used at postnatal day 10 (P10). Obese and diabetic C57BL/KsJ db/db (13–16 weeks old) and obese and normoglycemic C57BL/6 J ob/ob (13–16 weeks old) and respective age-matched lean control mice (db/+ and + /+) were obtained from the Garvan Institute breeding colonies. All animals were kept under conventional conditions on a 12 h light/dark cycle with free access to water and standard chow diet, except when otherwise stated. All animal procedures were performed in accordance with the National Institutes of Health guidelines and protocols were approved by the Swiss Research Councils and the Service de la Consommation et des Affaires Vétérinaires (SCAV) du canton de Vaud (License number VD3305) or by the Garvan Institute/St. Vincent’s Hospital Animal Experimentation Ethics Committee and the National Health and Medical Research Council of Australia.

### Rodent tissue panel and cell lines

RNA from mouse tissue panel were obtained from Amsbio. The murine insulinoma cell line MIN6B1^31^ was cultured in DMEM-Glutamax^TM^ medium (ThermoFisher) containing 25 mM glucose and 4 mM L-glutamine, and supplemented with 15% fetal calf serum (Gibco), 50 IU/mL penicillin, 50 ug/mL streptomycin and 70 µmol/L β-mercaptoethanol, while the rat INS 832/13 β-cell line^32^ was cultured in RPMI 1640 GlutaMAX^TM^ medium (ThermoFisher) containing 11 mM glucose and 2 mM L-glutamine, and supplemented with 10% fetal calf serum (Gibco), 10 mM Hepes pH 7.4, 1 mM sodium pyruvate and 50 µmol/L of β-mercaptoethanol.

### Pancreatic islet isolation and cell sorting

Rodent islets were isolated by collagenase digestion of the pancreas^33^ followed by Histopaque density gradient and hand-picking, to separate the islets from digested exocrine tissue. Prior to RNA extraction and *ex vivo* insulin secretion, islets were incubated in order to recover from the stress of the isolation for 2 h at 37 °C in RPMI 1640-GlutaMAX^TM^ medium (ThermoFisher) containing 3 mM glucose and 2 mM L-glutamine and supplemented with 10% fetal calf serum (Gibco), 10 mM HEPES, pH 7.4, 1 mM sodium pyruvate, 100 mg/mL streptomycin and 100 IU/mL penicillin. To obtain enriched fraction of α- and β-cells, Wistar rat islets were first dissociated into single cells by incubation in Ca^2+^/Mg^2+^ free phosphate buffered saline, 3 mM EGTA, and 0.002% trypsin (Gibco) for 5–7 min at 37 °C with constant gentle agitation. Islet cells were then separated by Fluorescence-Activated Cell Sorting (FACS) based on β-cell autofluorescence as previously described^34,35^. Islet cell enrichment was assessed by double immunofluorescence staining of α- (glucagon-positive) and β- (insulin-positive) sorted cells using mouse anti-glucagon (Abcam ab10988, 1:1000), guinea pig anti-insulin (Dako A0564, 1:100), goat anti-mouse IgG Alexa Fluor 555 (Thermo Fisher A-21422, 1:500), and goat anti-guinea pig IgG Alexa Fluor 488 (Thermo Fisher A-11073, 1:500) antibodies. On average, β-cell fraction contained 99.1 ± 0.9% of insulin-positive cells while α-cell fraction contained 88.8 ± 8.2% of glucagon-positive cells.

### RNA extraction and quantification

RNA was extracted from pancreatic islets using miRNeasy micro kit (Qiagen) followed by DNase treatment (Promega). One μg of RNA from different mouse tissue or 200 ng from islet RNA was reverse transcribed using the Quantitect RT kit (Qiagen) as described by the manufacturer. Real-time quantitative PCR reactions were assembled using FastStart Essential DNA green Master (Roche) and analyzed on Lightcycler 96 (Roche). To minimize the impact of differences in gene product abundance across different tissues, 3 reference genes (GAPDH, PolII and Actin) were used to normalize gene expression levels across the tissue panel. GAPDH is involved in glycolysis and is not a suitable reference in islets. We therefore used HPRT, that we have previously found to be a suitable refence gene in this tissue^35^, for islet gene expression analysis. Data were analyzed using the 2^–ʌʌC(T)^ method. Primer sequences are provided in Supplementary Table S1.

### Blood and plasma parameters

Blood was collected between 8AM and 10AM in both fed and 16h-fasted state. Glycaemia was determined from tail vein blood from awake animals using the Freestyle glucometer (Abbott, Switzerland). Plasma insulin was measured from blood samples collected by cardiac puncture (400ul) into heparinized tubes from euthanized 12 and 16 week-old mice. Insulin levels were determined by using an insulin enzyme immunoassay kit (Mercodia).

### Intraperitoneal glucose tolerance tests

Intraperitoneal glucose tolerance tests (IPGTT) were performed in the morning on 16 h fasted conscious mice by injecting intraperitoneally 2 mg/g body weight of warmed glucose (Sigma-Aldrich) solution dissolved in 0.9% NaCl. Tail blood samples (40ul) were collected in heparinized tubes at 0, 15, 30, 60 and 120 minutes after injection for measurement of blood glucose (glucometer, Abbott) and plasma insulin levels. Plasma insulin from IPGTT test was measured using the Ultrasensitive Mouse insulin ELISA kit (Crystal Chem, IL).

### *Ex vivo* glucose-induced insulin secretion

After 2 h recovery at 3 mM glucose in RPMI, freshly isolated P07Rik^flox/flox^ and P07Rik^+/+^ islets were distributed by batch of 25 in a 24-well plate, with 3 replicates per genotype for the insulin secretion and 1 for protein determination. Islets were then pre-incubated for 1 h at 37 °C in Krebs-Ringer bicarbonate buffer (KRBH) containing 25 mM HEPES, pH 7.4, 0.1% BSA (Sigma-Aldrich) and 2 mM glucose. After the pre-incubation period, the islets were first incubated for 45 min in KRBH at basal (2 mM) glucose level followed by another 45 min incubation at stimulated (16 mM) glucose concentration. Media were collected at the end of the two incubation periods and total insulin content of the islets was measured after acid-ethanol (0.2 mM HCl in 75% ethanol) extraction. Insulin release and content were determined by insulin Elisa kit (Mercodia) and total protein content by Bradford assay (BioRad).

### RNA sequencing and differential gene expression analysis

RNA was extracted from pancreatic islets of 3 pairs of 12 week-old male mutant and wildtype littermates using miRNeasy micro kit (Qiagen). Strand specific total RNA sequencing libraries were prepared using the NEB Ribo Zero protocol according to manufacturers’ instructions. Libraries were sequenced in an Illumina HiSeq 2000 to a depth of ~30 million 125 bp single-end reads. Reads mapping to rRNA in mouse (mm10) were discarded and the remaining reads were mapped to the mouse (mm10) genome using Hisat2 v2.1.0^36^. Expression counts for Ensembl v88 annotated genes were obtained using HTseq v0.9.1 (−minaqual = 1, otherwise default parameters)^37^. Differentially gene expression analysis was performed using DESeq. 2 v1.14.1^38^. Raw data was deposited in GEO under accession number GSE137389.

### Statistical analysis

Statistical significance was determined using parametric unpaired two-tailed Student’s t-test (Graph Pad Prism7). P values less than 0.05 (p < 0.05) were considered statistically significant.

## Supplementary information

Supplementary materials.

Supplementary Table 2.
