# Publisher Correction: Loss-of-function of the long non-coding RNA A830019P07Rik in mice does not affect insulin expression and secretion

**DOI:** 10.1038/s41598-020-65550-8

**Published:** 2020-05-15

**Authors:** Claudiane Guay, Baroj Abdulkarim, Jennifer Y. Tan, Gilles Dubuis, Sabine Rütti, David Ross Laybutt, Christian Widmann, Romano Regazzi, Ana Claudia Marques

**Affiliations:** 1 Department of Fundamental Neurosciences, University of Lausanne, Lausanne, Switzerland; 2 Department of Computational Biology, University of Lausanne, Lausanne, Switzerland; 3 Department of Physiology, University of Lausanne, Lausanne, Switzerland; 4 Present Address: Centre Européen d’Etude du Diabète, Strasbourg, France; 5 Garvan Institute of Medical Research, St. Vincent’s Clinical School, UNSW Sydney, Sydney, New South Wales Australia

Correction to: *Scientific Reports* 10.1038/s41598-020-62969-x, published online 14 April 2020

In the original version of this Article, the author David Ross Laybutt was incorrectly indexed. This error has now been corrected.

