# Supplementary material for: Loss-of-function of the long non-coding RNA A830019P07Rik in mice does not affect insulin expression and secretion
Source: Sci Rep. 2020 Apr 14;10:6413. doi: 10.1038/s41598-020-62969-x (PMC7156487; doi:10.1038/s41598-020-62969-x)

Loss-of-function of the long non-coding RNA A830019P07Rik in mice does not affect insulin expression and secretion

Claudiane Guay*[1], Baroj Abdulkarim*[2], Jennifer Y. Tan[2], Gilles Dubuis[3], Sabine Rütti[3,4], D. Ross Laybutt[5], Christian Widmann[3], Romano Regazzi[1] and Ana Claudia Marques[2]

[1] Department of Fundamental Neurosciences, University of Lausanne, Lausanne, Switzerland
[2] Department of Computational Biology, University of Lausanne, Lausanne, Switzerland
[3] Department of Physiology, University of Lausanne, Lausanne, Switzerland
[4] Current address : Centre Européen d'Etude du Diabète, Strasbourg, France.
[5] Garvan Institute of Medical Research,
St. Vincent's Clinical School, UNSW Sydney, Sydney, New South Wales, Australia
*Authors contributed equally to this study

Supplementary table S1: Primer Sequences

Primer name	Forward primer	Reverse primer
P07Rik Mouse qPCR	GCTGATTGCCCTCTAGCATTA	CAGCACCTACTAGTATCTGGGTGA
P07Rik rat exon 1	CAGACCTCCACGGCTTAAAA	GATACCGTTTTCCAGCCTCTC
P07Rik rat exon 2	GGAATGCGAAATGAGTCCCTA	ATTGCCTGACCTAGCCATTG
Actin mouse	GGCTGTATTCCCCTCCATCG	CCAGTTGGTACCAATGCCATGT
GAPDH mouse	TGTAGACCATGTAGTTGAGGTCA	AGGTCGGTGTGAACGGATTTG
Glucagon mouse	GAAGTTACCGCCCTGAGATT	CGCATTTATGACAAAGGGTTC
HPRT mouse/rat	AGTCCCAGCGTCGTGATTAG	AATCCAGCAGGTCAGCAAAG
Insulin2 mouse	GGCTTCTTCTACACACCCA	CAGTAGTTGTCCAGCTGGTA
A30019P07Rik WT loci	TGGGAGAAACAAAGACGGTG	TGTGCAAACATAACCCACTGC
A30019P07Rik mutant loci	TGGGAGAAACAAAGACGGTG	TCGTGGTATCGTTATGCGCC

Supplementary Figure S1: Example of genotyping PCR results

Schematic ((not drawn to scale) of the primer location and expected products within P07Rik (A) WT and (B) mutant loci. C) Agarose gel (2%) of products of genotyping multiplex PCR reaction for 15 individuals (1-15 top of the gel). Genotype is noted at the bottom of the lane as +/+, +/- and -/- for wild-type, heterozygous and homozygous P07Rik mutant.

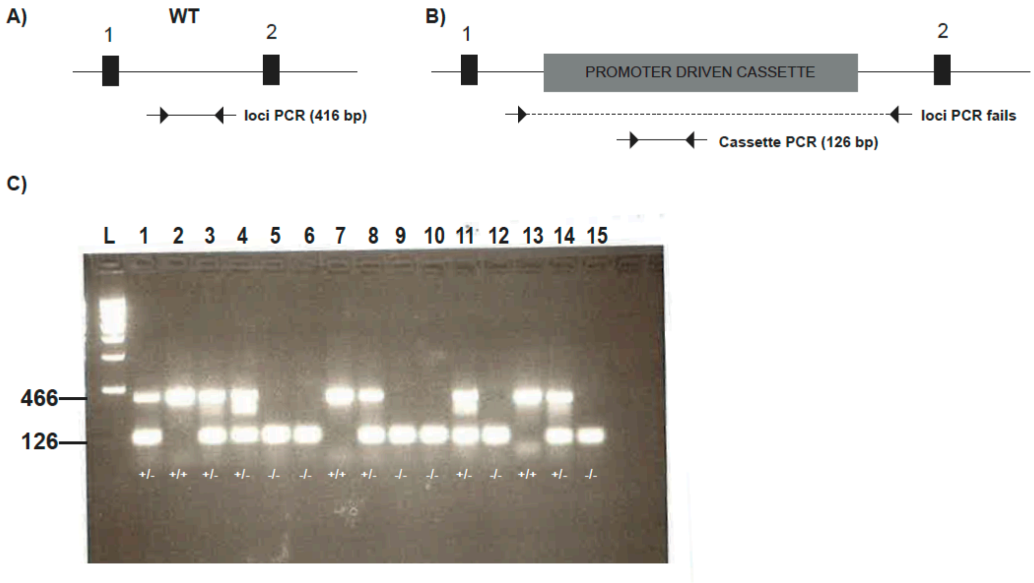

Supplement: Supplementary file 1 — Supplementary materials. [file 41598_2020_62969_MOESM1_ESM.pdf]
